# An Interaction between KSHV ORF57 and UIF Provides mRNA-Adaptor Redundancy in Herpesvirus Intronless mRNA Export

**DOI:** 10.1371/journal.ppat.1002138

**Published:** 2011-07-21

**Authors:** Brian R. Jackson, James R. Boyne, Marko Noerenberg, Adam Taylor, Guillaume M. Hautbergue, Matthew J. Walsh, Rachel Wheat, David J. Blackbourn, Stuart A. Wilson, Adrian Whitehouse

**Affiliations:** 1 Institute of Molecular and Cellular Biology, University of Leeds, Leeds, United Kingdom; 2 Astbury Centre for Structural Molecular Biology, University of Leeds, Leeds, United Kingdom; 3 Department of Molecular Biology and Biotechnology, University of Sheffield, Sheffield, United Kingdom; 4 School of Cancer Sciences, College of Medical and Dental Sciences, University of Birmingham, Birmingham, United Kingdom; University of California Berkeley, United States of America

## Abstract

The hTREX complex mediates cellular bulk mRNA nuclear export by recruiting the nuclear export factor, TAP, via a direct interaction with the export adaptor, Aly. Intriguingly however, depletion of Aly only leads to a modest reduction in cellular mRNA nuclear export, suggesting the existence of additional mRNA nuclear export adaptor proteins. In order to efficiently export Kaposi's sarcoma-associated herpesvirus (KSHV) intronless mRNAs from the nucleus, the KSHV ORF57 protein recruits hTREX onto viral intronless mRNAs allowing access to the TAP-mediated export pathway. Similarly however, depletion of Aly only leads to a modest reduction in the nuclear export of KSHV intronless mRNAs. Herein, we identify a novel interaction between ORF57 and the cellular protein, UIF. We provide the first evidence that the ORF57-UIF interaction enables the recruitment of hTREX and TAP to KSHV intronless mRNAs in Aly-depleted cells. Strikingly, depletion of both Aly and UIF inhibits the formation of an ORF57-mediated nuclear export competent ribonucleoprotein particle and consequently prevents ORF57-mediated mRNA nuclear export and KSHV protein production. Importantly, these findings highlight that redundancy exists in the eukaryotic system for certain hTREX components involved in the mRNA nuclear export of intronless KSHV mRNAs.

## Author Summary

Herpesviruses hijack cellular components to enhance viral gene expression. This is particularly important for the efficient nuclear export of herpesvirus intronless mRNAs to allow the production of viral proteins. We have previously demonstrated that Kaposi's sarcoma-associated herpesvirus encodes a conserved protein, ORF57, which recruits essential cellular mRNA export proteins onto the viral intronless mRNAs to form an export competent viral ribonucleoprotein particle. Specifically, we have shown that ORF57 interacts directly with the cellular export adaptor protein, Aly, to recruit other cellular mRNA export proteins. Surprisingly however, depletion of Aly has a limited effect on both cellular and viral mRNA nuclear export levels, suggesting a degree of redundancy in the export pathways and the existence of other export adaptor proteins. Here we have identified a novel interaction between ORF57 and a second export adaptor protein, UIF. We show for the first time that the ORF57-UIF interaction allows the recruitment of the essential cellular mRNA export proteins onto viral intronless mRNA, in cells lacking Aly. However, depletion of both Aly and UIF prevents the formation of an export competent viral ribonucleoprotein particle, suggesting that either Aly or UIF must be present for efficient KSHV intronless mRNA nuclear export and protein production.

## Introduction

Post-transcriptional events which regulate mRNA biogenesis are fundamental to the control of gene expression [1]. A nascent mRNA is therefore steered through multimeric RNA-protein complexes that mediate its capping, splicing, polyadenylation, nuclear export and ultimately its translation [2], [3]. A key aspect of these post-transcriptional events is that they are intrinsically linked [4]. For example, the act of splicing is coupled to the deposition of two distinct multiple protein complexes onto the mRNA which are involved in further processing events, namely the human transcription and export complex (hTREX) [5]–[7] and the exon-exon junction complex (EJC) [8]. The hTREX complex associates with the 5′end of the first exon by virtue of interactions with the cap-binding complex, and facilitates the nuclear export of the bulk mRNA through the TAP-mediated pathway [6]. In contrast, the EJC is deposited 20–24 nucleotides upstream of each exon-exon boundary and plays a role in mRNA surveillance [9] and translation enhancement [10]–.

The TREX complex is conserved from yeast to metazoans [3], [13], [14]. The human TREX complex comprises several core components: Aly (a NXF/TAP adaptor protein); UAP56 (a DEAD-box helicase); Tex1 (a protein of unknown function) and the stable multi-protein hTHO complex (hHpr1, hTho2, fSAP79, fSAP35 and fSAP24) [3]. Moreover, recent proteomic analysis has identified CIP29/Tho1 as a hTREX component that is conserved in both yeast and metazoans [15]. The precise mechanism of how hTREX is assembled onto the mRNA is not fully understood or characterised. UAP56 is thought to associate with mRNA at an early stage during the assembly of the spliceosome and functions to mediate the recruitment of Aly, CIP and the THO complex in an ATP-dependent manner to form hTREX [15], [16]. This involvement of the spliceosome in hTREX assembly reflects the splicing-dependent nature of mRNA nuclear export [16]–[18]. In addition to splicing, a functional 7-methylguanosine 5′ cap is also essential for hTREX recruitment, due to an interaction between Aly and the cap-binding complex protein, CBP80 [6]. Such cap-dependent recruitment of the export complex affords the mRNA polarity upon exiting the nuclear pore. Once assembled onto the mRNA, hTREX then instigates the recruitment of the nuclear export factor TAP, and its heterodimeric partner, p15, at the nuclear periphery, via a direct interaction with Aly [18]–[20]. TAP binding then elicits a RNA handover mechanism which results in the remodelling of the protein-mRNA interactions within the ribonucleoprotein complex [21]. Subsequently, TAP associates with the nucleoporins through central and carboxy-terminal domains, directing the ribonucleoprotein though the nuclear pore complex into the cytoplasm [22].

Surprisingly, considering the central role played by Aly in TAP recruitment, gene knockdown experiments performed in *Drosophila melanogaster* and *Caenorhabditis elegans* have shown that only UAP56, in contrast to Aly and THO-complex proteins, is required for bulk mRNA nuclear export [23]–[25]. Moreover, a genome-wide RNAi study in *D. melanogaster* reported that the conserved THO-complex was only required by a subset of transcripts for nuclear export [26], [27]. This data indicates a degree of redundancy is present in these pathways and suggests the existence of additional export adaptor proteins which are involved in bulk mRNA nuclear export. In support of this idea, a novel mRNA export adaptor protein has recently been identified that utilises the UAP56/TAP-mediated pathway. UAP56-interacting factor (UIF) was initially identified *in silica*, by virtue of sequence similarity to the characterised UAP56-binding domain found in Aly [28]. Notably, cellular expression levels of UIF appear to be linked *in vivo* to the relative expression of Aly, as miRNA-mediated depletion of Aly led to a dramatic increase in UIF expression. Importantly, simultaneous depletion of both Aly and UIF leads to a dramatic nuclear accumulation of bulk mRNA [28]. Therefore, it is believed that Aly and UIF bind independently to the same mRNA providing multiple export adaptor proteins to recruit multiple TAP molecules to ensure efficient mRNA nuclear export. Moreover, the observation that UIF expression increases in Aly-depleted cells is believed to be a redundancy mechanism that ensures cellular survival should Aly expression be compromised.

Given the importance of the formation of multimeric mRNA-protein complexes in mRNA biogenesis, it is not surprising that viruses manipulate and exploit these pathways. This is particularly important for herpesviruses which replicate in the host-cell nucleus and express numerous lytic intronless mRNAs. Due to the reliance of herpesviruses on the host cell machinery for efficient processing of their mRNAs, an immediate issue arises concerning the mechanism by which the viral intronless mRNAs are efficiently exported from the nucleus, given that the majority of cellular bulk mRNA nuclear export is intimately linked, and dependent upon, splicing [29]. We have investigated this potential roadblock to herpesvirus gene expression and replication in the gamma-2 herpesvirus, Kaposi's sarcoma-associated herpesvirus (KSHV) [30], which is associated with the AIDS-related malignancies Kaposi's sarcoma (KS), primary effusion lymphoma (PEL) and multicentric Castleman's disease [31]–[33]. To circumvent the roadblock of efficient intronless viral mRNA nuclear export, KSHV encodes a multi-functional protein termed ORF57/Mta. KSHV ORF57 is a functionally conserved protein found in all herpesviruses that plays a pivotal role in enhancing viral gene expression at a post-transcriptional level [34], [35]. ORF57 has been implicated in multiple steps of RNA biogenesis, including enhancing viral splicing, protecting viral RNAs from degradation to enhancing viral mRNA nuclear export and translation [36]–[39].

We have demonstrated that KSHV ORF57 promotes the nuclear export of intronless viral mRNAs via the TAP-mediated pathway, by directly interacting with the hTREX export adaptor, Aly [37]. Moreover, we investigated the composition and assembly of these export-competent intronless KSHV ribonucleoprotein particles (vRNP) and showed that ORF57 functions to recruit the complete hTREX complex to intronless viral mRNA, an event that is essential for viral intronless mRNA export and KSHV replication [37]. Furthermore, these properties are also conserved in other gamma-2 herpesvirus ORF57 homologues, such as the Herpesvirus saimiri (HVS) ORF57 protein [40], [41]. These data suggest that Aly is essential for ORF57-mediated KSHV intronless mRNA export, as well as playing an important role in mRNA nuclear export in other herpesviruses. However, experiments involving siRNA-mediated depletion of Aly report only a modest effect on ORF57-mediated KSHV intronless mRNA export, although only partial depletion of Aly was achieved [42]. This data correlates with depletion-related studies on the role of Aly in mRNA export in higher eukaryotes where, surprisingly, Aly has been shown to be dispensable in mRNA export [23], [24]. Similar stories are also evident for other herpesviruses mRNA export proteins. For example, an observed interaction between ICP27 (the HSV-1 ORF57 homologue) and Aly was initially reported as important for HSV-1 mRNA export [43]. However, subsequent functional studies using siRNA-mediated depletion of Aly led to the authors suggesting that Aly is not essential for ICP27-mediated HSV-1 mRNA export [44]. This suggests that additional cellular mRNA export proteins play important roles in herpesvirus intronless mRNA export. Indeed, recently it has been demonstrated that the SR proteins, SRp20 and 9G8, can contribute to efficient export of herpes simplex virus 1 mRNAs [45].

Herein we report a novel interaction between the KSHV ORF57 protein and the recently identified mRNA export adaptor protein, UIF. Moreover, we provide data to suggest that ORF57 may preferentially bind Aly compared to UIF. Furthermore, we investigate whether the linked expression of UIF and Aly plays a role in the apparent redundancy of Aly in herpesvirus intronless mRNA nuclear export. We provide the first evidence that the ORF57-UIF interaction enables the recruitment of the complete hTREX and the nuclear export factor, TAP, to KSHV intronless mRNA in Aly-depleted cells. Strikingly, we demonstrate that depletion of both Aly and UIF inhibit the formation of an ORF57-mediated nuclear export competent ribonucleoprotein particle and consequently prevent ORF57-mediated nuclear export of intronless viral mRNAs and KSHV protein production. Importantly, these findings highlight that redundancy exists in the eukaryotic system for certain hTREX components involved in the mRNA nuclear export of intronless KSHV mRNAs.

## Results

### KSHV ORF57 interacts with the UAP56 interacting protein, UIF

**Figure 1:**
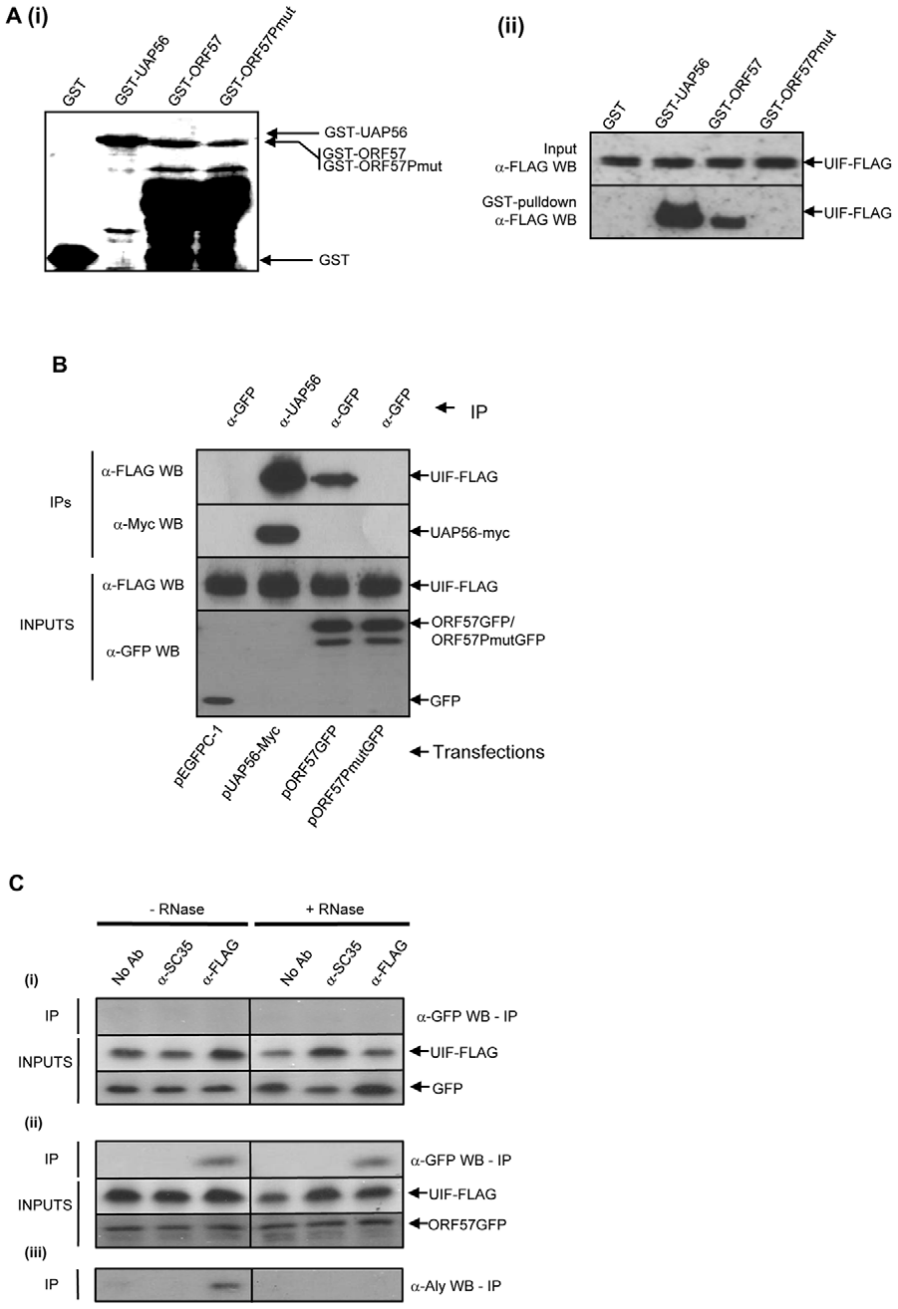
KSHV ORF57 interacts directly with UIF. (A) (i) Bacterially expressed GST-, GST-UAP56-, GST-ORF57- and GST-ORF57Pmut-bound to glutathione agarose beads and separated by SDS-PAGE, proteins were visualised by coomassie staining. (ii) GST Pull-down assays were performed using pUIF-FLAG transfected cell lysates. Precipitated UIF protein was detected by western blot analysis using a FLAG-specific antibody. UIF-FLAG transfected cell lysate served as a loading control (Input). (B) Immunoprecipitations using GFP- or UAP56-specific antibodies were performed using cell lysates cotransfected with UIF-FLAG in the presence of either pEGFP, pUAP56-Myc, pORF57GFP or pORF57PmutGFP. Precipitated UIF-FLAG, UAP56-myc, GFP, ORF57GFP and ORF57PmutGFP were detected by western blot analysis using antibodies specific to FLAG, myc or GFP. Transfected cell lysates served as a loading control (Inputs). (C) Immunoprecipitations were performed in the presence or absence of RNase, using a no antibody control, (SC35)- or FLAG-specific antibodies on cell lysates cotransfected with pUIF-FLAG in the presence of either (i) pGFP and (ii) pORF57GFP. Precipitated proteins were detected by western blotting using GFP- or FLAG-specific antibodies. (iii) ORF57-transfected co-immunoprecipitations were also immunoblotted with an Aly-specific antibody to confirm the activity of the RNase digestion to abolish the RNA-dependent interaction between Aly and UIF.

KSHV ORF57 interacts directly with the cellular export adaptor protein Aly to recruit cellular hTREX, comprising UAP56 and the hTHO complex, onto a viral intronless mRNA to form an export competent ribonucleoprotein particle [37]. However, ORF57 and homologues can mediate nuclear export of an intronless viral mRNA in Aly-depleted cells [42], suggesting that alternative export pathways may be targeted by the ORF57 protein. Therefore, to determine whether ORF57 interacts with alternative export adaptor proteins, GST-pulldown and co-immunoprecipitations assays were performed to assess if ORF57 interacted with the recently identified UAP56 interacting protein, UIF. Initially, recombinant GST-, GST-UAP56 or GST-ORF57 fusion proteins were produced and used in GST-pulldown assays. It must be noted however, that although full length GST-ORF57 is produced, a large proportion of the product is degraded as previously observed [37]. GST-pulldown experiments were therefore performed using equal amounts of total protein from each GST construct immobilised to beads followed by incubation with 293T cell lysates transfected with pUIF-Flag. Analysis showed that UIF interacted with both UAP56 and KSHV ORF57 (Figure 1A). To confirm these results co-immunoprecipitation experiments were also performed. 293T cells were transfected with either pEGFP, pUAP56-myc or pORF57GFP in the presence of pUIF-Flag and used in co-immunoprecipitation experiments with GFP or UAP56-specific antibodies. Results confirmed the interaction between UIF and KSHV ORF57 (Figure 1B).

We have previously identified an ORF57 mutant protein, ORF57PmutGFP, which is unable to interact with Aly and therefore recruit the remainder of the hTREX complex onto viral intronless mRNAs [37]. Moreover, we demonstrated that this mutant is unable to efficiently export viral intronless mRNA from the nucleus suggesting that the recruitment of a complete hTREX complex is required for ORF57-mediated nuclear export. ORF57PmutGFP contains site-directed alterations of two proline residues within a PxxP poly-proline motif, situated in the previously identified minimal Aly-binding domain encompassing residues 181–215. We have previously demonstrated that although ORF57PmutGFP is unable to bind Aly, it still retains features of the wild type ORF57 protein, namely localising to nuclear speckles, the ability to homodimerise, bind KSHV RTA and bind intronless viral mRNA [37]. To assess whether this mutant could interact with UIF, GST-pulldown experiments and co-immunoprecipitation experiments were performed as described above using GST-ORF57Pmut and pORF57PmutGFP, respectively. In both cases the mutant ORF57 protein, which fails to bind Aly, also lacks the ability to interact with UIF (Figure 1A and 1B). Importantly, this suggests that the failure of ORF57PmutGFP to recruit hTREX and efficiently export intronless viral mRNAs from the nucleus may be due to the inability to bind either Aly or UIF.

To determine if the interaction between ORF57 and UIF depended on RNA bridging, co-immunoprecipitation experiments were repeated in the absence and presence of RNase. 293T cells were transfected with either pEGFP or pORF57GFP in the presence of pUIF-Flag and co-immunoprecipitation assays were performed using a polyclonal Flag-specific antibody. In addition, no antibody and a negative control antibody (α-SC-35) were also used in the analysis. ORF57 was readily precipitated using the Flag-specific antibody in contrast to negative controls. Moreover, the observed interaction was still detected in the presence of RNase suggesting the interactions are not due to RNA bridging (Figure 1C). To ensure the RNase treatment was effective the immunoprecipitations were also blotted with an Aly-specific antibody. Results show that the UIF-Aly interaction is RNA dependent as previously described [28], [36].

**Figure 2:**
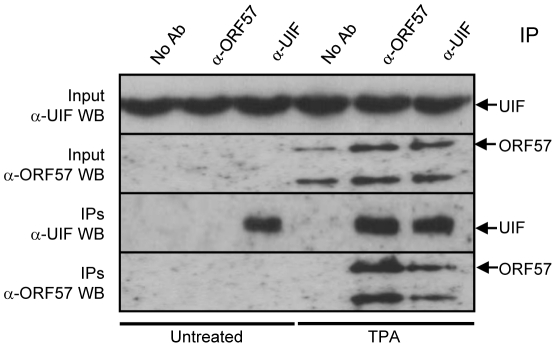
ORF57 interacts directly with UIF during KSHV lytic replication. Immunoprecipitations were performed on unreactivated or reactivated BCBL-1 cells using no antibody control, ORF57- or UIF-specific antibodies. Precipitated proteins were detected by western blot using UIF- and ORF57-specific antibodies. BCBL-1 cells were reactivated by the addition of TPA (20 ng/ml).

In order to address potential overexpression artefacts of the above co-immunoprecipitation experiments and also determine whether ORF57 interacts with UIF during KSHV lytic replication, latently-infected BCBL-1 cells remained uninduced or reactivated using the phorbol ester, TPA. Lytic expression was confirmed by the detection of ORF57 using Western blot analysis in the reactivated samples (Figure 2). Uninduced and reactivated cell lysates were then incubated with no antibody control, ORF57- or UIF-specific antibodies. Reciprocal western blot analysis using the antibodies in reverse demonstrated that ORF57 interacts with UIF during KSHV lytic replication (Figure 2). Therefore, these data provide the first evidence of a viral protein associating with UIF.

**Figure 3:**
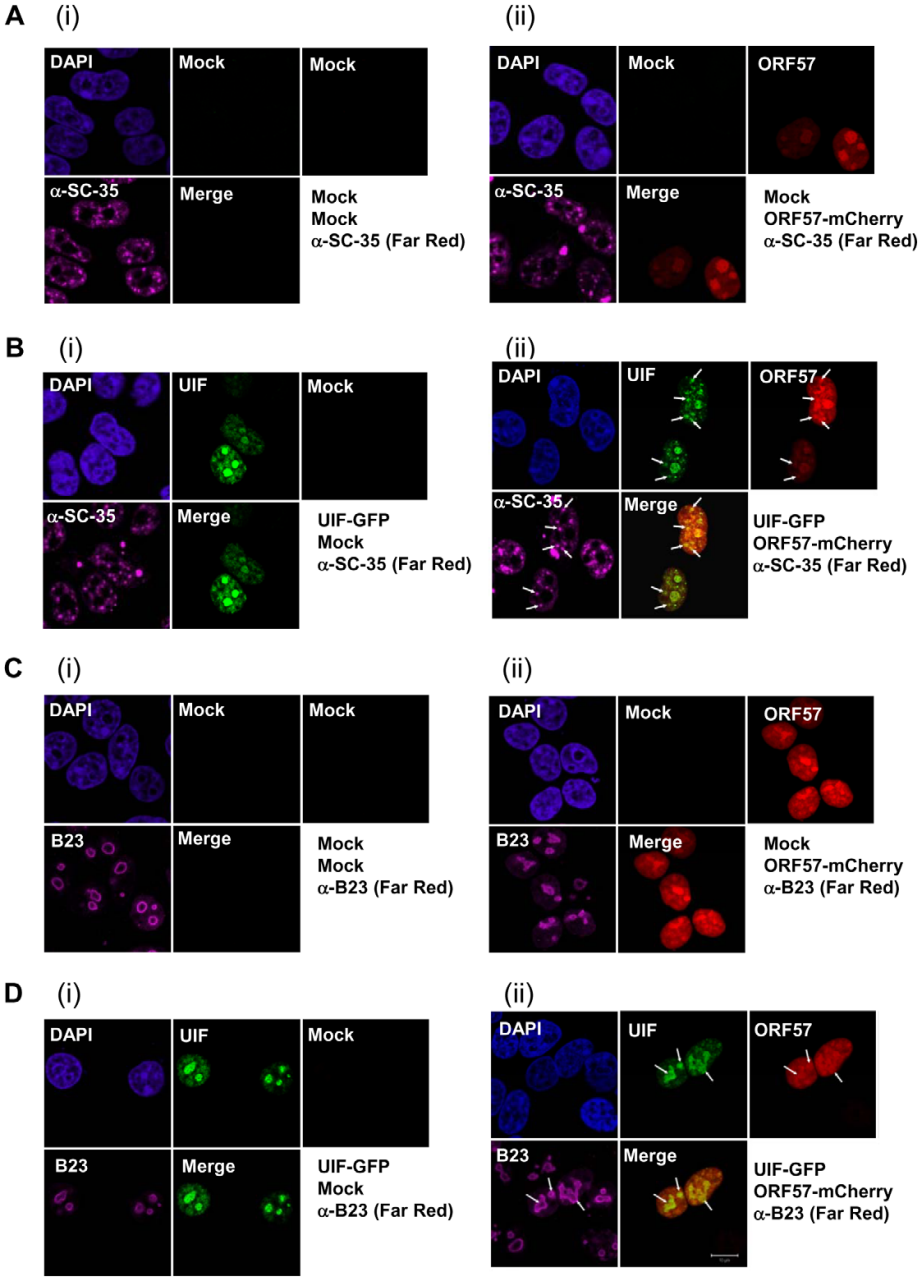
KSHV ORF57 colocalises with UIF in the nucleus and nucleolus. 293T cells were either mock (Ai, Ci), or transfected with pORF57-mCherry (Aii, Cii) or pUIF-GFP (Bi, Di) and in combination (Bii, Dii), incubated for 24 h, fixed and immunofluoresence staining performed. ORF57 and UIF were visualised by direct fluorescence of Cherry and GFP, respectively. Subcellular localisation within the nuclear speckles or nucleolus was confirmed using antibodies specific to SC-35 (A, B) or B23 (C, D), respectively. A merge of the mCherry/GFP channels is also included for all images. White arrows indicate nuclear speckles (Bii) or nucleolar localisation (Dii).

ORF57 is a nucleocytoplasmic protein that is predominately observed in the nucleus, specifically colocalising with nuclear speckle and nucleoli-associated proteins [42], [46]. Therefore, we were interested to determine whether ORF57 colocalises with UIF in either of these subnuclear domains. To this end, 293T cells were cultured on poly-L lysine coated coverslips and transfected with either pORF57-mCherry or pUIF-GFP alone or in combination. The subcellular localisation of ORF57 and UIF were observed via direct fluorescence, in addition indirect-immunofluorescence was performed to identify nuclear speckles and the nucleolus using SC35- (Figure 3Bii) and B23- (Figure 3Dii) specific antibodies, respectively. As previously observed ORF57 colocalises with both subnuclear domain markers (Figure 3Bii and 3Dii). Moreover, UIF was also found to localise with these subnuclear structures and also colocalises with the ORF57 protein (Figure 3B and 3D). However, results demonstrate that the majority of ORF57 and UIF colocalise in the nucleolus whereas only a proportion of ORF57 and UIF colocalise with the nuclear speckle marker, SC35.

### ORF57 facilitates the loading of UIF onto KSHV intronless mRNAs

**Figure 4:**
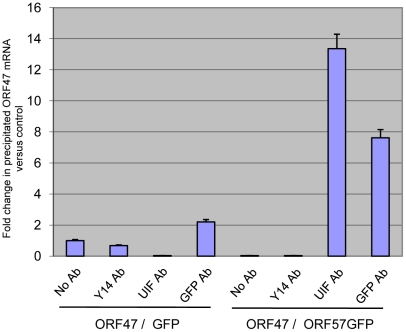
ORF57 is required for the recruitment of UIF to a KSHV intronless mRNA. RNA immunoprecipitations were performed in 293T cells cotransfected with KSHV pORF47 in the presence of either pEGFP or pORF57GFP. After UV crosslinking cell lysates were immunoprecipitated using GFP- or UIF-specific antibodies. In addition, no antibody and a negative control Y14 antibody were also used as controls. Protein was then digested, and immunoprecipitated RNA was analysed by qRT-PCR.

One major difference between the mRNA export adaptor proteins Aly and UIF is the mechanism they utilise to be loaded onto mRNA. Aly has been shown to associate with mRNA in a UAP56 and splicing-dependent manner [47], in contrast UIF is loaded onto mRNA via the histone chaperone FACT [28]. We have previously demonstrated that ORF57 is required for the recruitment of Aly and the remainder of the hTREX complex onto viral intronless mRNAs, therefore we were intrigued to determine if UIF could associate with intronless viral mRNAs in an ORF57-independent manner using RNA-immunoprecipitation (RNA-IP) assays. A vector expressing KSHV ORF47 (a late structural intronless gene) was transfected into 293T cells with either pEGFP or pORF57GFP. Total cell lysates were then used in immunoprecipitations performed with either No, Y14- (negative control), UIF- or GFP-specific antibodies and the amount of ORF47 precipitated was measured by qRT-PCR. RNA-IPs performed on cell extracts transfected with pORF47 and pEGFP failed to show an interaction between UIF and the viral intronless ORF47 mRNA (Figure 4). In contrast, extracts from cells transfected with both pORF47 and pORF57GFP displayed a clear interaction between UIF, ORF57GFP and the intronless viral ORF47 mRNA (Figure 4). These data show that although UIF can associate with cellular spliced and unspliced single exon cellular mRNAs, ORF57 is required for the recruitment of UIF onto intronless viral mRNA.

### UIF links ORF57 to the hTREX component, UAP56

**Figure 5:**
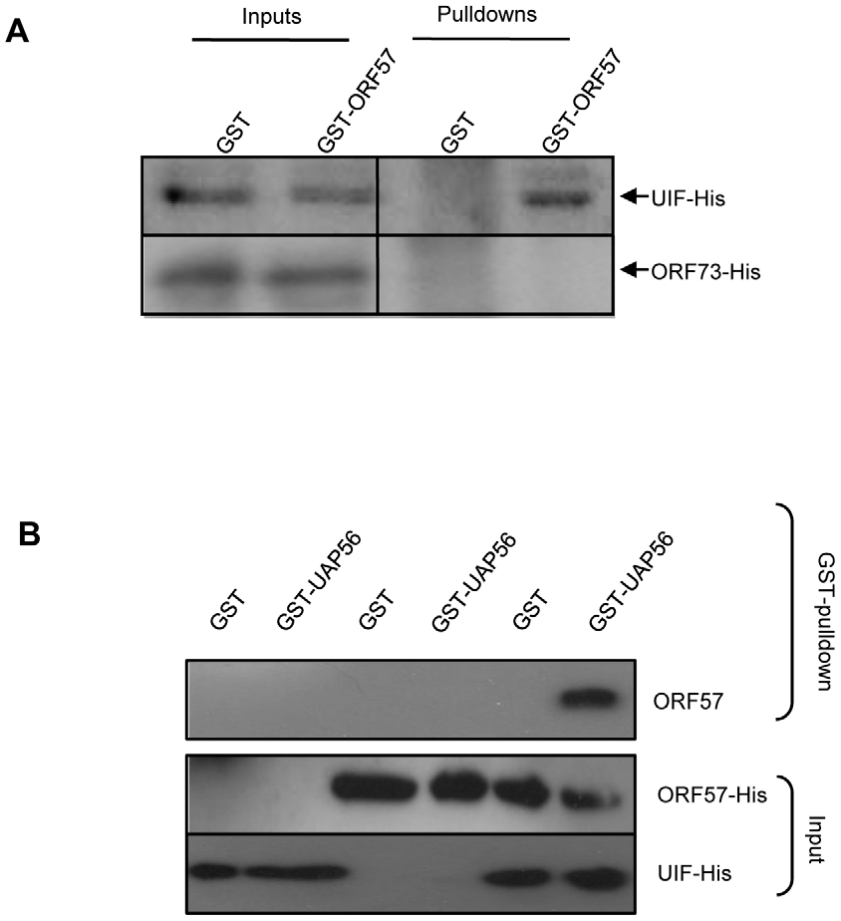
ORF57 is linked to the hTREX complex by UIF; however, ORF57 preferentially interacts with Aly over UIF. (A) Recombinant GST, and GST-ORF57 were bound to glutathione agarose beads and GST Pull-down assays performed using purified recombinant UIF-His or control ORF73-His proteins. Precipitated proteins and inputs were analysed by western blotting using a His-specific antibody. (B) Reconstitutive GST pulldowns were performed using GST or GST-UAP56 bound to glutathione agarose beads and incubated with recombinant purified ORF57-His or Aly-His alone or in combination. Precipitated proteins and inputs were analysed by western blotting using a His-specific antibody.

We have previously demonstrated that the nuclear export adaptor protein, Aly, is recruited to viral intronless mRNAs in a splicing-independent manner by directly interacting with ORF57. Once bound it then leads to the recruitment of the remaining components of hTREX, in turn leading to efficient nuclear export of these viral intronless mRNAs via a TAP-mediated pathway [37]. We therefore next sought to determine if UIF can perform a similar function by linking ORF57 to hTREX components such as UAP56. Initially, we determined whether ORF57 interacted with UIF directly using GST-pulldown assays. Recombinant GST- and GST-ORF57 proteins were immobilised to beads and incubated with purified recombinant UIF-6xHis or a negative control purified recombinant HVS ORF73-6xHis protein [48]. UIF-6xHis was precipitated by GST-ORF57 but not the negative GST control, moreover ORF73-6xHis failed to interact with either GST or GST-ORF57 (Figure 5A). These data provide further support for the direct interaction between ORF57 and UIF.

Given the fact that ORF57 and UIF interact directly, we next determined whether UIF can bridge the interaction between ORF57 and hTREX components, such as UAP56, which we have previously shown fails to interact with ORF57 directly [37]. Reconstitutive GST-pulldowns were therefore performed using recombinant GST- and GST-UAP56 proteins immobilised to beads and incubating with either purified recombinant ORF57-6xHis or purified recombinant UIF-6xHis alone or in combination. No interaction with GST or GST-UAP56 was observed in the presence of ORF57-6xHis alone. In contrast, an interaction between GST-UAP56 and ORF57 was observed in the presence of purified UIF-6xHis protein (Figure 5B), suggesting that UIF can facilitate the formation of the ORF57-hTREX complex. This provides the first evidence to demonstrate that UIF could function to assemble the hTREX complex on viral intronless mRNAs.

### ORF57 may preferentially bind Aly over UIF

**Figure 6:**
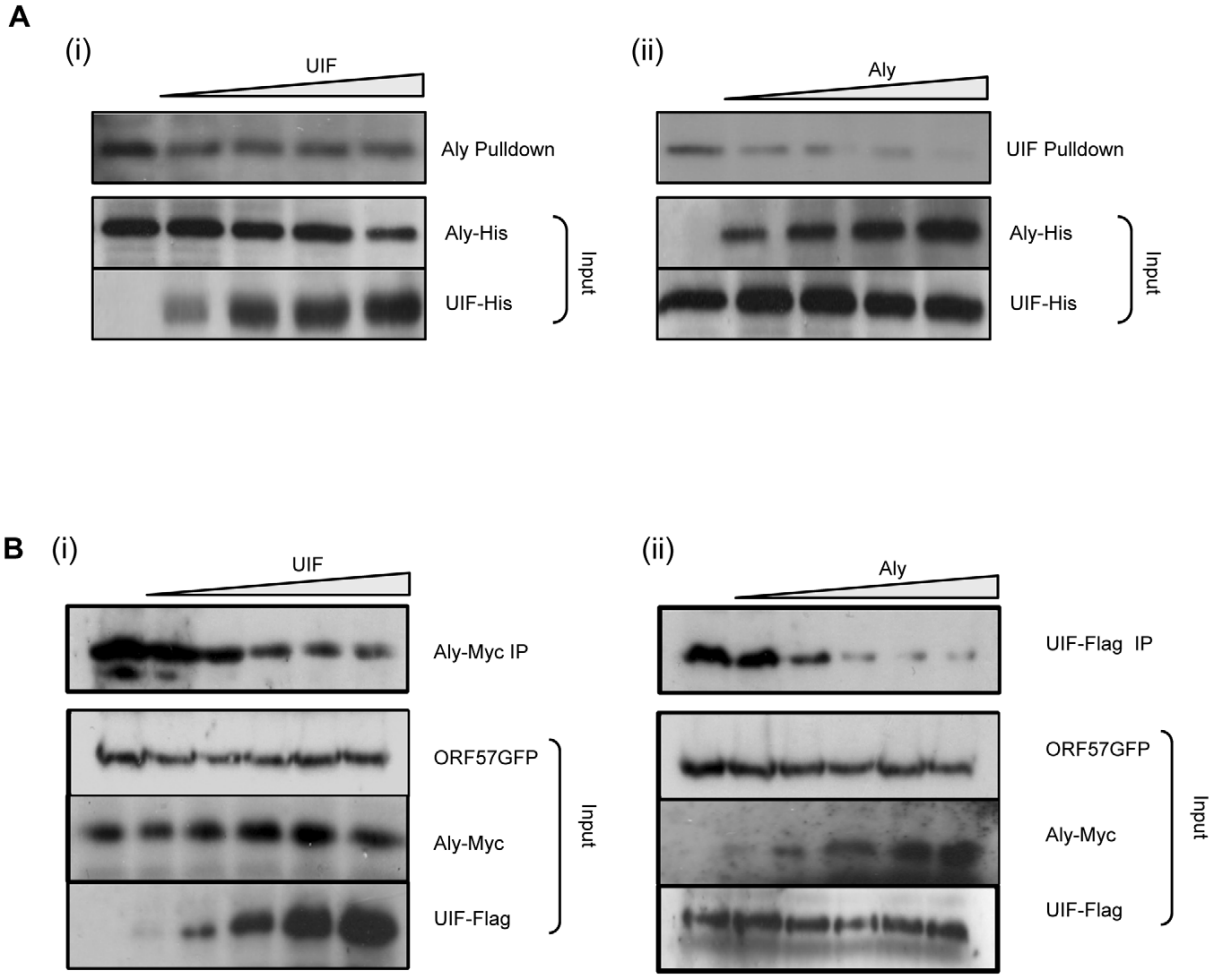
KSHV ORF57 may preferentially bind Aly over UIF. (A) Competition assays were performed using recombinant GST-ORF57 bound to glutathione agarose beads and incubated with (i) a constant amount of purified Aly-His (1 µg) and increasing amounts of purified UIF-His (0, 0.5, 1, 2, 3 µg), (ii) a constant amount of purified UIF-His (1 µg) and increasing amounts of purified Aly-His (0, 0.5, 1, 2, 3 µg), Precipitated protein and inputs were analysed by western blotting using a His-specific antibody. (B) Dose-dependent co-immunoprecipitations were performed using 293T cells cotransfected with (i) 0.5 ug of pORF57GFP and 0.5 ug of pAly-myc, in addition to increasing amounts (0, 0.1, 0.5, 0.8, 1.2 ug) of pUIF-Flag or (ii) 0.5 ug of pORF57GFP and 0.5 ug of pUIF-Flag, in addition to increasing amounts (0, 0.1, 0.5, 0.8, 1.2 ug) of pAly-myc, empty vector was also added to ensure a similar amount of DNA was transfected in each sample. After 24 hours, cell lysates were incubated with GFP-TRAP-Affinity agarose beads and the amount of precipitated (i) Aly or (ii) UIF was identified by immunoblotting with Myc- or Flag-specific antibodies, respectively. Western blots for input loading are also shown for ORF57, Aly and UIF constructs.

The above data demonstrate that UIF interacts directly with ORF57 and suggest that it can function to bridge an interaction between ORF57 and the remaining hTREX components. This mechanism is similar to our previous observations regarding the functional significance of the Aly-ORF57 interaction, and therefore leads to the intriguing question of whether ORF57 has a preference for Aly binding over UIF or vice versa. To address this question we performed competitive GST pulldown assays. Recombinant GST-ORF57 protein was immobilised to beads and incubated with a constant amount (1 µg) of purified recombinant Aly-6xHis protein, in addition the pulldown was spiked with increasing amounts of purified recombinant UIF-6xHis protein (0, 0.5, 1, 2, 3 µg). Western blot analysis was then performed using an Aly-specific antibody. Results demonstrate that the binding of Aly to GST-ORF57 is only slightly reduced in the presence of increasing amount of UIF (Figure 6Ai), suggesting that UIF cannot out-compete Aly for ORF57 binding. Similar spiked experiments were performed using a constant amount of UIF and increasing amounts of Aly. In contrast, results showed that even low quantities of Aly led to a dramatic loss of UIF binding to the ORF57 protein (Figure 6Aii). These results reveal that Aly can out-compete UIF for ORF57 binding, suggesting that ORF57 may preferentially bind Aly to form an export competent ribonucleoprotein particle.

However as shown in Figure 1A, although bacterial expression of full length GST-ORF57 results in a full length ORF57 protein, a large proportion of degraded products are also produced. Therefore, to further assess the possibility that ORF57 may interact with Aly preferentially over UIF, dose-dependent coimmunoprecipitation assays were performed. To this end, 293T cells were cotransfected with 0.5 ug of pORF57GFP and 0.5 ug of pAly-myc, in addition to increasing amounts of pUIF-Flag (0, 0.1, 0.5, 0.8, 1.2 ug). After 24 hours, cell lysates were incubated with GFP-TRAP-Affinity agarose beads and the amount of precipitated Aly was identified by immunoblotting with a Myc-specific antibody. Results again show that the binding of Aly is only slightly reduced in the presence of increasing amounts of UIF (Figure 6Bi). Moreover, reciprocal dose-dependent coimmunoprecipitations were performed using 0.5 ug of pORF57GFP and 0.5 ug of pUIF-Flag, in addition to increasing amounts of pAly-myc (0, 0.1, 0.5, 0.8, 1.2 ug). In contrast, results suggest that higher concentrations of Aly can significantly reduce the amount of precipitated UIF (Figure 6Bii). These results corroborate the above GST pulldown assays and suggest that ORF57 may preferentially bind Aly over UIF to form an export competent ribonucleoprotein particle.

### Depletion of both Aly and UIF is required to inhibit ORF57-mediated virus ribonucleoprotein particle formation

**Figure 7:**
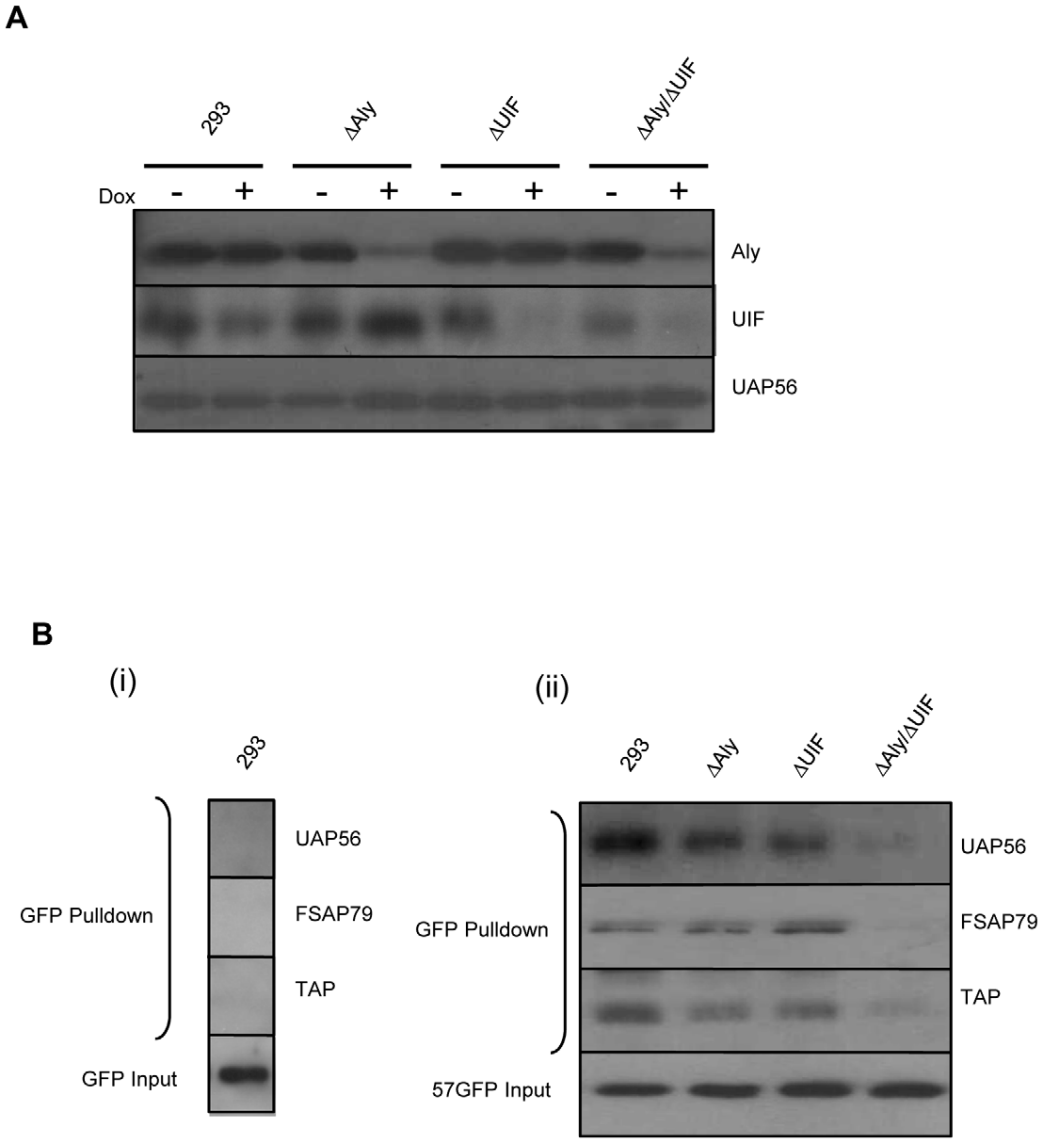
Knockdown of both Aly and UIF impairs the ability of ORF57 form an export competent viral ribonucleoprotein particle. (A) 293, 293ΔAly, 293ΔUIF and 293ΔAlyΔUIF cells were mock treated or treated with 2 µg/ml doxycyclin grown for 72 h. Cell lysates were analysed by western blotting using Aly-, UIF- and UAP56-specific antibodies. (B) (i) 293 cells were transfected with pEGFP and grown for a further 24 h. Cell lysates were incubated with GFP-TRAP-Affinity beads and after washing, the precipitated proteins were detected by western blotting using GFP-, UAP56-, FSAP79- and TAP-specific antibodies. (ii) 293, 293ΔAly, 293ΔUIF and 293ΔAlyΔUIF cells were treated with 2 µg/ml doxycyclin and grown for 48 h before being transfected with pGFP-ORF57 and grown for a further 24 h. Cell lysates were incubated with GFP-TRAP-Affinity beads and after washing, the precipitated proteins were detected by western blotting using GFP-, UAP56-, FSAP79- and TAP-specific antibodies. Transfected cell lysates were used as an input control.

Having established that both Aly and UIF can bridge an interaction between ORF57 and hTREX components, such as UAP56, we next sought to determine the effect of depleting Aly and UIF either singularly, or in combination, on the ability of ORF57 to form an export competent ribonucleoprotein particle containing the complete hTREX complex and the nuclear export factor TAP. To this end, we have utilised doxycycline inducible 293 cell lines expressing miRNAs targeting Aly, UIF or both Aly and UIF [28]. Effective depletion of Aly, UIF or both proteins can be observed after 72 hours post doxycycline induction (Figure 7A). However, a caveat of this type of experiment is that depletion of multiple mRNA export factors in combination may firstly be toxic to the host cell and second inhibit the expression of ORF57 itself as recently reported [49]. Characterisation of the cell viability and growth of the cells depleted with both Aly and UIF has previously been performed and results show they are viable and grow for 4 days post knockdown prior to cell death at day 6 [28]. Therefore all experiments using these cell lines were performed in this 4 day window. Moreover, to ensure ORF57 protein production, cells were transfected at 48 hours prior to complete Aly or UIF depletion at 72 hours.

To assess viral ribonucleoprotein particle formation, wild type 293 cells and each miRNA-targeted cell line were induced with doxycycline to deplete the respective proteins and after 48 hours' induction, each cell line was transfected with pORF57GFP. After a further 24 hours when maximum Aly and UIF depletion has occurred, cell lysates were used in co-immunoprecipitation experiments using GFP-TRAP-Affinity agarose beads. Western blot analysis was then performed using UAP56-, FSAP79- (a hTHO complex component) and TAP-specific antibodies. As a negative control, GFP was also transfected into the wild type 293 cell line and co-immunoprecipitations performed using GFP-TRAP-Affinity agarose beads, no interactions were observed with any of the hTREX components or TAP. However, results showed that expression of ORF57 in the wild type 293 cell line led to the precipitation of UAP56, FSAP79 and TAP suggesting that ORF57 expression leads to the formation of an export competent ribonucleoprotein particle (Figure 7B). Similar complex formation was observed in cell lines depleted singularly for Aly and UIF, where ORF57 can precipitate UAP56, FSAP79 and TAP (Figure 7B). In contrast, depletion of Aly and UIF in combination significantly reduced the interaction between ORF57 and the hTREX components and the nuclear export factor TAP. Importantly, these data demonstrate that either Aly or UIF are required for the formation of an ORF57-mediated nuclear export competent ribonucleoprotein particle.

### Either Aly or UIF is required for ORF57-mediated mRNA nuclear export and KSHV protein production

**Figure 8:**
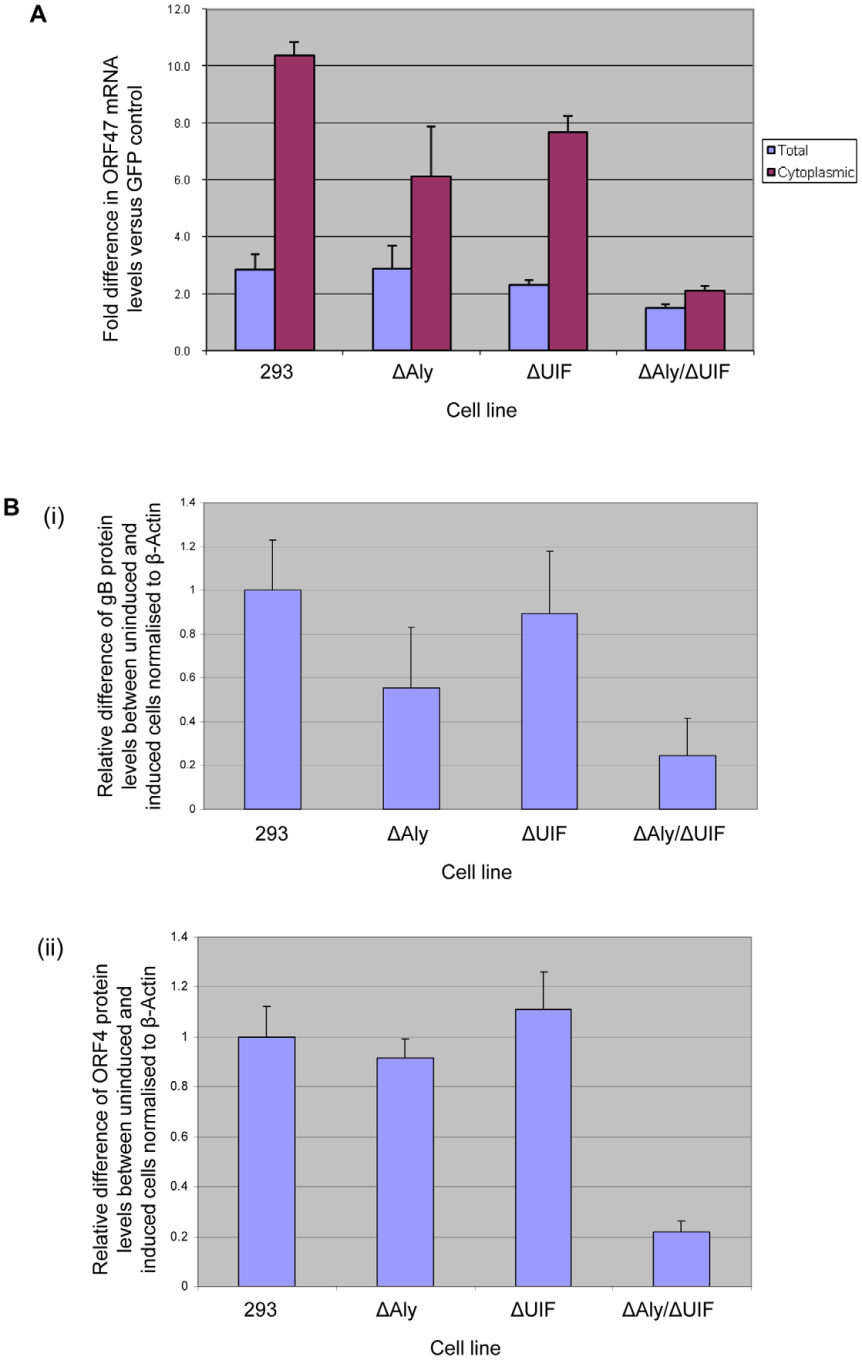
Knockdown of both Aly and UIF impairs the ability of ORF57 to export KSHV intronless mRNA from the nucleus and reduces KSHV late protein production. (A) 293, 293ΔAly, 293ΔUIF and 293ΔAlyΔUIF cells were treated with 2 µg/ml doxycyclin and grown for 48 h before being transfected with pORF47 in the presence of either pEGFP or pGFP-ORF57. 24 h post-transfection RNA was isolated from total and cytoplasmic fractions and relative levels were analysed by qRT-PCR using GAPDH as a reference. Fold increase was determined by ΔΔcT and statistical significance by a non-paired *t*-test. Data from 3 independent experiments is presented as fold increase versus pGFP-transfected controls. (B) 293, 293ΔAly, 293ΔUIF and 293ΔAlyΔUIF cells were mock treated or treated with 2 µg/ml doxycyclin and grown for 48 h before infection with KSHV at a MOI = 1. At 48 h post-infection cells lysates were analysed by western blot using antibodies specific to KSHV (i) gB and (ii) ORF4 proteins. Results are shown of densitometry analysis of the western blots from 3 independent experiments carried out using the ImageJ software and is shown as expression of gB or ORF4 between uninduced and induced cell lines relative to β-actin.

The above data suggest that ORF57 must interact with either export adaptor protein, Aly or UIF, to recruit hTREX and the nuclear export protein TAP, to form an export competent ribonucleoprotein particle. Therefore, we next determined whether both UIF and Aly were required for efficient ORF57-mediated nuclear export of viral intronless mRNAs. To this end, we assessed the ability of ORF57 to enhance the nuclear export of the KSHV intronless ORF47 mRNA, using a previously described assay to compare the accumulation of ORF47 mRNA in the cytoplasm [46]. Essentially, cells are transfected with a plasmid expressing the intronless KSHV ORF47 gene in addition to either GFP or wild type ORF57 constructs. After 24 hours, RNA is extracted from total and cytoplasmic fractions and RNA levels quantified using qRT-PCR. Total RNA levels are assessed to ensure similar expression levels of the ORF47 mRNA in each sample, where an increase in cytoplasmic levels of ORF47 mRNA signifies an increase in ORF57-mediated mRNA export levels. Therefore, to assess the ability of ORF57 to export ORF47 mRNA from the nucleus in the absence of either UIF or Aly or both, wild type 293 cells and each miRNA-targeted cell line were induced with doxycycline to deplete the respective proteins and after 48 hours induction, each cell line was transfected with pORF57GFP and pORF47. Again, this allowed sufficient time to express ORF57 prior to optimal export adaptor protein depletion. After a further 24 hours, RNA was extracted from total and cytoplasmic fractions and ORF47 levels assessed by qRT-PCR. Results demonstrated that ORF47 mRNA levels from total cell fractions are similar in wild type and the depleted cell lines. Moreover, in the control 293 cell line ORF47 mRNA accumulates in the cytoplasm in the presence of ORF57 as previously described [46]. Similarly, mRNA can accumulate in the cytoplasm of cells depleted singularly for Aly and UIF, however, a reduction in export efficiency was observed of approximately 40% and 23% of wild type levels, respectively (Figure 8A). In contrast, depletion of both Aly and UIF together led to a dramatic reduction of ORF47 mRNA accumulation in the cytoplasm with an 80% decrease compared to wild type levels (Figure 8A).

We next tested whether the observed reduction in the ability of ORF57 to export intronless mRNAs from the nucleus in cell lines depleted for Aly and UIF had any effect on KSHV protein production. To this end, the wild type 293 cells and each miRNA-targeted cell line were induced with doxycycline to deplete the respective proteins and after 48 hours induction, each cell line was infected with recombinant KSHV at a MOI = 1. This time point was used to allow sufficient time to express ORF57 prior to optimal export adaptor protein depletion. After a further 48 hours, the cell lysates were analysed by immunoblotting using KSHV glycoprotein B- and ORF4-specific antibodies. Results showed that gB protein expression in cell lines singularly depleted for either Aly or UIF was reduced by ∼42% and ∼10%, respectively, whereas little or no reduction was observed for ORF4 protein levels in the singularly depleted cells. Strikingly, however depletion of both Aly and UIF led to a dramatic reduction in both gB and ORF4 expression levels of 78% and 79%, respectively (Figure 8B). These results suggest that depletion of UIF has limited if any effect of virus replication, however, depletion of UIF together with Aly had a dramatic negative effect on KSHV protein production. However, it must be noted that this reduction in protein levels may also stem from altered levels of one or more key cellular proteins involved in KSHV lytic protein production.

Taken together, our data suggest that either one of the cellular nuclear export adaptor proteins, Aly or UIF, is required for the formation of an ORF57-mediated nuclear export competent ribonucleoprotein particle which is essential for KSHV protein production.

## Discussion

The nuclear export of bulk mRNA is mediated by the conserved heterodimeric export receptor, TAP/p15 [3]. Cellular mRNAs gain access to TAP/p15 via interaction with a group of RNA-binding proteins termed export adaptors. The first mRNA export adaptor to be identified in higher systems was Aly/REF, and subsequent work from a number of groups led to the current model where Aly is recruited to the 5′ cap of spliced mRNA along with several other proteins to form a multimeric protein complex termed hTREX [6]. The hTREX complex facilitates the association of bound mRNAs with TAP/p15 thus licensing nuclear export. In addition to Aly, several other hTREX components have been identified including the DEAD-box helicase UAP56, hTex1, the multi-protein THO complex and recently, CIP29 [15]. While the underlying mechanism of hTREX-mediated mRNA export is loosely understood, the individual functions of the hTREX components remain elusive.

Perhaps the greatest enigma surrounding TAP/p15-mediated mRNA export is the apparent redundancy that exists for certain hTREX proteins. This is particularly true for Aly, where a number of different studies have shown that the metazoan homologue, REF1, is not required for the bulk export of mRNA [23], [24]. These studies suggest that additional mRNA export adaptors must exist which can function to link nascent mRNA to the TAP/p15 heterodimer. Moreover, this raises the intriguing possibility that, via the use of numerous different mRNA export adaptor proteins, a further layer of control may exist to regulate gene expression. Indeed, several recent reports have highlighted that differences exist within component members of mRNA export complexes associated with different classes of mRNAs. For example, HSP70 mRNA only requires Aly and the co-adaptor Thoc5 to mediate TAP recruitment [50]. Moreover, an alternative mRNA export (AREX) complex, distinct to hTREX has recently been identified which comprises the related RNA helicase URH49, instead of UAP56 [51]. Interestingly, each helicase regulates a specific set of mRNAs associated with distinct subsets of key mitotic regulators. In addition, members of the SPEN family of proteins, RBM15 and OTT3 are functionally similar, in that they can bind RNA and physically interact with TAP. However, the association of OTT3 with TAP is attenuated compared to RBM15, leading to speculation that strong and weak variants exist that may function during developmental or tissue specific mRNA processing events [52]. These data galvanise the hypothesis that ultimately it is the recruitment of TAP/p15 that is required for nuclear export, and that one function of the export adaptor proteins is to provide selectivity to this system. Such a hypothesis is consistent with, and offers an explanation to, conflicting data regarding the nuclear export of KSHV intronless mRNAs.

Herpesviruses hijack the TAP/p15-mediated mRNA export pathway in order to enhance the nuclear export of viral intronless mRNA. We have previously shown that during KSHV replication the virus-encoded ORF57 protein procures the hTREX complex (and subsequently TAP/p15) via a direct interaction with Aly, facilitating the efficient export of KSHV intronless mRNAs [37]. We proposed therefore, that as the ORF57-Aly interaction provides the link between the virus mRNA and hTREX, it was likely that Aly would be essential for KSHV mRNA export. This hypothesis was supported by data showing that an ORF57 mutant, ORF57PmutGFP, unable to bind Aly was no longer functional in virus mRNA export. However, similarly to previous studies in *D. melanogaster* and *C. elegans*, siRNA-mediated depletion of Aly did not translate to a decrease in ORF57-mediated nuclear export of KSHV intronless mRNA, although only partial knockdown of Aly was observed [42]. Correspondingly, the HSV homologue of ORF57, ICP27, was shown to directly interact with Aly. Moreover, studies in *Xenopus laevis* oocytes showed ICP27 dramatically stimulated the export of intronless viral mRNAs, and a mutant ICP27 protein that failed to interact with REF is inactive in viral mRNA export [43]. Again however, siRNA-mediated depletion of Aly has been shown not to affect HSV-1 mRNA export [44].

Herein, we demonstrate that redundancy exists in the eukaryotic system for certain hTREX components involved in the mRNA nuclear export of intronless KSHV mRNAs. Evidence for such redundancy in export adapter proteins was recently provided by the identification of a second mRNA export adaptor protein, UIF [28]. Importantly, cellular expression levels of UIF appear to be linked *in vivo* to the relative expression of Aly, as depletion of Aly leads to a dramatic increase in UIF expression. This would therefore account for the modest reduction in mRNA nuclear export in Aly-depleted cells. Indeed, as shown in Figure 1 and 5, ORF57 interacts directly with UIF and thus is able to recruit hTREX/TAP/p15 allowing efficient intronless virus mRNA nuclear export in Aly-depleted cells (Figure 8).

Recent analysis has also suggested that additional mechanisms exist to ensure the nuclear export of viral transcripts in other herpesviruses. For example, ICP27 can bind directly to TAP, suggesting ICP27 can bypass nuclear export adapter proteins [53]. However, although analysis of ICP27 mutants unable to interact with TAP export showed greatly reduced intronless viral mRNA export, it was not completely abolished suggesting other cellular proteins may have a role. Indeed, recent analysis has shown that nuclear accumulation of HSV-1 mRNA is reduced when cells were treated with siRNAs specific for the SR proteins, SRp20 and 9G8, confirming that other cellular export proteins, such as SR proteins, can contribute to HSV-1 mRNA nuclear export [45]. Similarly, the EBV ORF57 homologue, SM/EB2, can interact with SRp20, although to date, this interaction has been implicated in alternative splicing mechanisms [54]. However, EBV SM/EB2 has been previously shown to interact with alternative cellular export factors, such as CRM-1 [55]. An alternative approach may be employed by the hCMV ORF57 homologue, UL69, which interacts with other hTREX proteins required for bulk mRNA nuclear export, such as UAP56 [56]. However, current work is ongoing to determine if these homologues interact with UIF. Moreover, the role of UIF may also have wider implications in the field of virology. Influenza A virus produces capped and polyadenylated mRNAs in the nucleus of infected cells that resemble mature cellular mRNAs, which require export by the TAP-mediated pathway [57]. Depletion of Aly had little effect on viral mRNA export, but reduction of UAP56 levels strongly inhibited trafficking and/or translation of influenza mRNAs [58]. It will now be interesting to determine whether UIF also substitutes for Aly function in this viral system.

There are however, some important mechanistic differences between Aly and UIF which have implications for KSHV intronless mRNA nuclear export. The hTREX component, CIP29, bridges the Aly-UAP56 interaction to form a trimeric complex that is assembled in an ATP-dependent manner [15]. Importantly, the recruitment of Aly to the mRNA requires an interaction with the 5′ cap and is dependent on splicing [6]. However, UIF appears to be co-transcriptionally loaded onto burgeoning mRNAs via an interaction with the histone chaperone, FACT [28]. It appears therefore that Aly and UIF are deposited onto the same mRNA separately and independently, a hypothesis supported by ribonuclease-treated co-immunoprecipitation experiments, which show that the interaction between Aly and UIF is facilitated by RNA-bridging [28], [36]. These data suggest that there are two distinct cellular mechanisms that can each recruit TAP to an mRNA. This raises a number of interesting questions with regards to how ORF57 orchestrates the recruitment of hTREX (and ultimately TAP/p15) via UIF. As seen in Figure 4, UIF is recruited to KSHV intronless mRNA only in the presence of ORF57, this is in stark contrast to the mechanism by which UIF is loaded onto cellular mRNA. Why UIF is not loaded onto KSHV intronless transcripts via FACT is unclear. One possible explanation is that FACT does not interact with RNA polymerase II during the transcription of ORF47 mRNA in this assay, possibly due to incomplete chromatinisation of vector DNA. Alternatively, recruitment of UIF to both spliced and unspliced mRNA maybe partially dependent on UAP56 and we have previously shown that UAP56 recruitment to KSHV mRNA is dependent on the ORF57 protein [37].

As mentioned above, Aly and UIF are loaded separately onto the same cellular mRNA via different mechanisms and both function to ultimately recruit TAP/p15 to the mRNA via interactions with hTREX. Intriguingly, we show in Figure 6, that ORF57 may preferentially bind to Aly over UIF, using both competitive GST-pulldown and dose-dependent coimmunoprecipitation assays. Why KSHV ORF57 would evolve to preferentially bind Aly over UIF is at present uncertain. One possibility is that Aly is the major export adaptor protein and UIF forms a backup or default pathway. This is not without precedent as proteins expression levels suggest that Aly is more abundantly expressed than UIF and UIF protein levels significantly increase in Aly-depleted cells [28]. Alternatively, it is possible that ORF57 may have a higher affinity for Aly due to important functional differences in how the Aly export adaptor recruits the remaining hTREX components to virus mRNA, compared with UIF. Alternatively, Aly and UIF could recruit different components of the hTREX complex to a KSHV mRNA, highlighted by the Aly-specific recruitment of CIP29, and that the export of KSHV intronless mRNA is more reliant on these Aly-recruited hTREX proteins.

As discussed earlier, a number of siRNA-mediated studies have proposed that Aly is not essential for KSHV intronless mRNA export. However, we have previously described an ORF57 mutant protein, ORF57Pmut, which is unable to interact with Aly and failed to export viral intronless mRNAs [37]. The region mutated in ORF57Pmut maps to a PxxP motif in the N-terminal region of the protein. It is not known whether the PxxP motif mutated in ORF57Pmut is a direct interaction site for Aly, or if this mutant confers some structural change of ORF57 in the Aly binding region. Importantly, herein we have shown that this mutant is also unable to interact with UIF, suggesting that ORF57Pmut is ‘dead’ with regards to export adaptor interaction. This explains therefore why this mutant is unable to export viral intronless mRNAs, as it is unable to bind to either Aly or UIF (Figure 1). This result is also confirmed by depletion of both these export adaptors which lead to a block in KSHV mRNA nuclear export. Importantly, Aly depletion in these and previous studies have shown that UIF expression is increased and therefore UIF probably replaces Aly as the dominant export adaptor protein. It is tempting to speculate that the link between increased UIF expression in Aly-depleted cells is a redundancy mechanism that ensures cellular survival should Aly expression be compromised.

The fact that ORF57Pmut is unable to interact with both Aly and UIF would suggest that the PxxP motif is either the complete ORF57 interacting motif, or part of the interacting motif, for Aly and UIF binding, and that the binding sites for the two proteins are either identical or overlap to some degree. Alternatively, the PxxP motif may cause a loss of interaction of both Aly and UIF by altering the structure of each of the binding sites. Importantly, our competition assays demonstrate that ORF57 may preferentially bind to Aly over UIF. These observations suggest that Aly and UIF may compete for a binding site on ORF57, and further studies are now required to determine if this is the case. Interestingly, we have recently identified the key residues that interact directly with Aly in both HSV-1 ICP27 and herpesvirus saimiri (HVS) ORF57 using solution-state NMR and mapped this interaction to a WRV/A motif [59]. Due to the sequence differences between ORF57 homologues this motif does not appear in KSHV ORF57, although the region of KSHV ORF57 that interacts with Aly has been mapped to the N terminus (aa 1–215). We are currently investigating the interacting residues for both Aly and UIF within this N-terminal region of KSHV ORF57 using solution-state NMR.

In summary, our results demonstrate the first known interaction between a viral protein and the newly described export adaptor protein, UIF. Importantly, the ORF57-UIF interaction is sufficient to recruit the hTREX complex onto viral intronless mRNAs and highlights that redundancy exists in the eukaryotic system for certain hTREX components involved in the mRNA nuclear export of intronless KSHV mRNAs. It now seems clear that the events which lead up to TAP/p15 recruitment to the mRNA are not linear. Indeed, it appears that multiple pathways exist by which an mRNA can bind TAP/p15 and be licensed for nuclear export. The existence of numerous export adaptor proteins may partly be explained in terms of redundancy but there is strong evidence to suggest that this also generates specificity within the system.

## Materials and Methods

### Plasmid and antibody details

Details of oligonucleotides used for qRT-PCR have been described previously [37], [46]. KSHV, hTREX and UIF-related plasmid constructs have been described previously [6], [28], [37]. KSHV ORF57- and ORF4- specific antibodies were a kind gift from Gary Hayward (Johns Hopkins, Baltimore) and Brad Spiller (Cardiff University), respectively. Antibodies against SC-35, Flag, Myc and Aly (Sigma), GFP and mCherry (Clontech), B23 (Santa Cruz), KSHV gB (Abcam) and GAPDH (Abcam) were purchased from their respective suppliers. Western blot analysis was carried out using specific antibodies at 1∶1000 dilution, except for UIF-specific antibody (1∶250) and GFP-specific antibody (1∶5000). Antibodies used for immunofluorescence studies were at a dilution of 1∶250.

### Cell culture, viruses and transfection

293 inducible cells lines which specifically deplete Aly, UIF and both Aly and UIF have been previously reported [28]. They were produced using the FLP-In T-REX 293 cells (Invitrogen) system to express miRNAs to each specific export adapter protein, miRNA sequences are detailed in Hautbergue et al., 2009. HEK-293T cells, HEK-293T BAC36 cells harbouring a recombinant KSHV BAC36 genome and FLP-In T-REX 293 cells were cultured in Dulbecco's modified Eagle medium (DMEM, Invitrogen) supplemented with glutamine, 10% foetal calf serum (FCS, Invitrogen) and penicillin-streptomycin. 293T BAC36 cells were reactivated using TPA (20 ng/ml) for the designated time. miRNA expression from FLP-IN T-REX 293 cells was induced with 2 µg/ml doxycyclin (Sigma) for the designated time. Plasmid transfections were carried out using Lipofectamine 2000 (Invitrogen) or GeneJuice (Novagen) and were carried out as per the manufacturer's instructions. rKSHV.219 (KSHV) was produced from the latently infected Vero line [60]. This virus specifies red fluorescent protein (RFP) from the KSHV lytic PAN promoter, green fluorescent protein (GFP) from the EF-1α promoter, and encodes a puromycin resistance gene. Vero cells stably infected with rKSHV.219 were maintained in MEM Eagles medium, 2.2 g/L NaHCO_3_, 10% fetal calf serum, puromycin (5 ug/ml) (Sigma-Aldrich, Poole, UK) and penicillin and streptomycin (Invitrogen). To induce KSHV lytic replication in these cells, they were infected with BacK50, a baculovirus construct encoding the lytic switch RTA protein, and treated with 1.25 mM sodium butyrate (Sigma). 48 h after KSHV reactivation, the supernatant was harvested, centrifuged (500*g*, 15 mins) to remove cell debris, and the virions concentrated by centrifugation (65,000*g*, 4 h). The virion pellet was resuspended overnight in EBM2 medium (Lonza, Clonetics). The rKSHV.219 titre was determined on 293 cells, quantifying GFP-positive cells by fluorescence microscopy. 293 and 293 derived cells were infected with KSHV. To this end, cells were plated at 1.25×10^5^ cells per well in 24-well plates for infection and cultured overnight. The culture medium was then removed and replaced with virus diluted in EBM2 basal media after 24 hrs. Cells were then centrifuged for 30 min at 420× g at room temperature. Cells were transferred to a 37°C incubator (5% CO_2_, humidified) for 90 min. Virus supernatant was removed and cells were washed once in cell culture media and incubated for 48 hrs before being harvested.

### Expression and purification of recombinant proteins

Recombinant GST, GST-ORF57, GST-ORF57pmut, GST-UAP56 and UIF-His, Aly-His and ORF73-His were expressed and purified as previously described [36],[37],[61]. Purification of Baculovirus recombinant ORF57-6xHis was as per the manufacturer's instructions (Invitrogen) using the pFASTBac protocol.

### In vitro pull-down assays and immunoprecipitation assays

GST pull-down experiments and co-immunoprecipitations were performed as described previously [62], [63]. GFP-TRAP-Affinity (Chromotek) experiments were performed as per the manufacturer's instructions. RNA immunoprecipitation experiments were carried out as follows: 1×10^7^ adherent 293T cells were transiently transfected with appropriate GFP-containing plasmid DNA. After the appropriate amount of time cells were washed in ice-cold PBS and UV irradiated (900 mJ/cm^2^) using a Stratalinker 2400 (Stratagene) to crosslink protein and RNA. Cells were then scraped, transferred to an RNA-free tube and pelleted at 300× g for 3 min. Cell pellets were then resuspended in 2 ml lysis buffer [Dulbecco's PBS, 1% Nonidet P-40 (v/v), 1 µl/ml RNaseOUT (Invitrogen), 1× Complete EDTA-free Protease inhibitor (Roche)]. Cells were left on ice for 30 min before being centrifuged for 10 min at 15,000× g. The clear lysate was then transferred to a clean RNA-free tube. 1 ml of the cleared lysate was added to 30 µl pre-washed GFP-TRAP-Affinity agarose beads (Chromotek) per IP and immunoprecipitated at 4°C with end-over-end mixing for 4 hrs. Beads were washed 3 times in ice-cold PBS containing 1× Complete EDTA-free protease inhibitor (Roche) followed by a further 2 times in PBS. Beads where then incubated in protease buffer (Dulbecco's PBS, 1% Nonidet P-40 (v/v), 0.1% SDS (w/v), 0.5 mg/ml Proteinase K) for 30 min at 50°C. RNA was extracted using TRIzol reagent (Invitrogen) as per the manufacturer's directions. cDNA was then produced from 10 µl of RNA using Superscript II RT (Invitrogen) and qPCR performed to analyse the relative levels of cDNA. RT-ve samples were used as controls.

### GST pull-down competition assays

Bacterially expressed GST-tagged ORF57 was immobilised to GST beads and used for GST pulldown competition assays. Recombinant His-tagged Aly or UIF was expressed and purified as previously described [36], [37]. Equal amounts of Aly-His (1 µg) were used in the pull-downs with increasing amounts of UIF-His (0, 0.5, 1, 2, 3 µg). The converse experiments were also performed with equal amounts of UIF-His (1 µg) and increasing amounts of Aly-His (0, 0.5, 1, 2, 3 µg).

### Real-time qRT-PCR

To assess ORF57-mediated ORF47 mRNA export efficiency, 293T and inducible cells were cotransfected with ORF47 and ORF57 expression constructs. After 24 hours RNA was extracted from total and cytoplasmic fractions using TRIzol (Invitrogen) as described by the manufacturer. Cytoplasmic fractions were produced by lysis of cells in 200 µl of PBS 1% Triton-X 100(v/v) containing 40 U of RNaseOUT (Invitrogen), prior to TRIzol purification. RNA was DNase treated using the Ambion DNase-free kit, as per the manufacturer's instructions, and RNA (1 µg) from each fraction was reverse transcribed with SuperScript II (Invitrogen), as per the manufacturer's instructions, using oligo(dT) primers (Promega). 10 ng of cDNA was used as template in SensiMix*Plus* SYBR qPCR reactions (Quantace), as per manufacturer's instructions, using a Rotor-Gene Q 5plex HRM Platform (Qiagen), with a standard 3-step melt program (95°C for 15 seconds, 60°C for 30 seconds, 72°C for 20 seconds). With GAPDH as internal control mRNA, quantitative analysis was performed using the comparative C_T_ method as previously described [46].

### Immunofluorescence

Immunofluorescence staining and visualisation by microscopy was carried out as previously described [64]. Visualisation was performed on an LSM 510 Meta confocal microscope (Zeiss) and images were analysed using the LSM imaging software (Zeiss).
